# Prognostic significance of interim PET/CT response for the treatment of advanced-stage marginal zone lymphoma in the post-rituximab era

**DOI:** 10.1038/s41598-020-68310-w

**Published:** 2020-07-15

**Authors:** Ga-Young Song, Sang Eun Yoon, Seok Jin Kim, Jin Seok Kim, Youngil Koh, Joon-Ho Moon, Sung Yong Oh, Ho Sup Lee, Ho-Jin Shin, Young Rok Do, Won Sik Lee, Dae sik Kim, Yong Park, Ho-Young Yhim, Deok-Hwan Yang

**Affiliations:** 1 Chonnam National University Hwasun Hospital, Hwasun, Jeollanam-do Republic of Korea; 2 Jeonbuk National University Medical School, Jeonju, Jeollabukdo Republic of Korea; 3 Research Institute of Clinical Medicine of Jeonbuk National University-Biomedical Research Institute of Jeonbuk National University Hospital, Jeonju, Republic of Korea; 4 Sungkyunkwan University School of Medicine, Seoul, Republic of Korea; 5 Yonsei University College of Medicine, Seoul, Republic of Korea; 6 Seoul National University Hospital, Seoul, Republic of Korea; 7 Department of Hematology/Oncology, Kyungpook National University Hospital, Kyungpook National University School of Medicine, Daegu, Republic of Korea; 8 Dong-A Medical Center, Busan, Republic of Korea; 9 Kosin University Gospel Hospital, Busan, Republic of Korea; 10 Division of Hematology-Oncology, Department of Internal Medicine, Medical Research Institute, Pusan National University Hospital, Pusan, Republic of Korea; 11 Keimyung University Dongsan Medical Center, Daegu, Republic of Korea; 12 Busan Paik Hospital, Inje University, Busan, Republic of Korea; 13 Korea University Guro Hospital, Seoul, Republic of Korea; 14 Division of Hematology/Oncology, Department of Internal Medicine, Korea University School of Medicine Anam Hospital, Seoul, Republic of Korea; 15 Department of Internal Medicine, Jeonbuk National University Medical School, 20 Geonji-ro, Deokjin-gu, Jeonju, 54907 Republic of Korea; 16 Department of Hematology/Oncology, Chonnam National University Hwasun Hospital, School of Medicine, Chonnam National University, 322 Seoyang-ro, Hwasun-eup,, Hwasun-gun, Jeollanam-do 58128 Republic of Korea

**Keywords:** B-cell lymphoma, Outcomes research

## Abstract

There are still controversies about the use of interim positron emission tomography/computed tomography (PET/CT) in indolent non-Hodgkin lymphoma due to the variable fluorodeoxyglucose (FDG) avidity. Therefore, this study aimed to evaluate the roles of interim PET/CT in marginal zone lymphoma (MZL), a representative indolent lymphoma. We analyzed the data of 146 MZL patients. All were treated with rituximab-containing immunochemotherapy. Interim PET/CT scan was performed after 2–3 cycles of therapy, and the response was assessed using the Deauville 5-point scales (5-PS) and a semi-quantitative assessment using the SUVmax reduction rate (ΔSUVmax). Progression-free survival (PFS) was well stratified according to a visual assessment of interim PET/CT using 5-PS (*p* < 0.001). Particularly, there was a significant difference in PFS between patients with interim score 1–2 and those with score 3. However, ΔSUVmax did not predict the survival outcome using 59.8% of the optimal cutoff value. In the multivariate analysis, failure to achievement of grade 1–2 in interim PET/CT was significantly associated with inferior PFS (HR, 2.154; 95% CI 1.071–4.332; *p* = 0.031). The interim PET/CT response based on the 5-PS is useful for predicting PFS of patients with MZL in the post-rituximab era.

## Introduction

The clinical significance of fluorine-18-fluorodeoxyglucose (^18^F-FDG) positron emission tomography/computed tomography (PET/CT) in the diagnosis or management of lymphoma is gradually becoming important^1,2^. Although it remains controversial, several previous studies reported that the interim PET/CT response could predict long-term clinical outcomes in cases of Hodgkin lymphoma (HL), aggressive non-Hodgkin lymphoma (NHL), and follicular lymphoma^3–5^. However, the role of ^18^F-FDG PET/CT assessment in indolent lymphomas other than follicular lymphoma is unclear due to variable ^18^F-FDG avidity^6^.

Marginal zone lymphoma (MZL), which develops in the marginal zone or edge of the lymphoid tissue, comprises 7–8% of all B-cell NHL cases worldwide. In Korea, MZL is 2nd most common mature B-cell lymphoma (23.0%) because of the relatively lower incidence of follicular lymphoma and chronic lymphocytic leukemia/small lymphocytic leukemia than those in the Western population^7,8^. MZL has a heterogenous subgroup classified as extranodal, splenic and nodal. Extranodal MZL, also known as mucosa-associated lymphoid tissue (MALT), constitutes the majority (~ 70%) of MZL cases and has a variable clinical presentation depending on the involved sites^9^. There is no standard recommended treatment for MZL, but rituximab-containing immunochemotherapy is considered appropriate treatment for advanced-stage cases^10,11^. The ^18^F-FDG avidity of MALT lymphoma, which is known 40–95%, varies according to the extranodal involvement sites, histologic features and morphologic features such as tumor size^12–18^. Several previous studies suggested potential clinical relevance of ^18^F-FDG PET/CT in the diagnostic evaluation of MZL^12,19^. In the latest National Comprehensive Cancer Network guidelines update, performing ^18^F-FDG PET/CT scan was essentially suggested in the initial staging work up of MZL regardless of subtypes^20^. However, evidence is still lacking regarding the use of PET/CT for treatment response assessments in cases of MZL.

The prognostic significance of ^18^F-FDG PET/CT scan for patients with MZL has been suggested in a study by Kim and colleagues^21^. In this study, patients who achieved complete response on the interim and/or post-treatment ^18^F-FDG PET/CT showed higher 5-year progression-free survival (PFS) rates than those with non-CR (post-treatment: 54.2% vs. 0.0%, *P* = 0.003; interim: 62.5% vs. 15.6%, *P* = 0.026)^21^. However, they assessed the PET scan using binary classification of metabolic response, but didn’t use current standard response assessment tool, Deauville criteria using 5-point scale (5-PS), or semi-quantitative assessment using SUVmax reduction rate (ΔSUVmax). Therefore, the present study aimed to evaluate the prognostic impact of interim ^18^F-FDG PET/CT assessment according to the Deauville 5-PS score (DS) and ΔSUVmax on survival outcomes of patients with MZL who were treated with rituximab-containing immunochemotherapy.

## Patients and methods

### Patients and study design

This multicenter, retrospective study was designed to evaluate the prognostic impact of interim ^18^F-FDG PET/CT response on the survival outcomes of patients with MZL. The clinical data of 178 adult (> 19 years of age) patients with newly diagnosed MZL from 13 independent institutions were initially analyzed between January 2008 and January 2018. Seven patients who were not administrated rituximab-based immunochemotherapy and one patient who did not receive any treatment were excluded. In addition, 24 patients who did not undergo initial or interim ^18^F-FDG PET/CT assessments were also excluded. A total of 146 patients were included in the final analysis.

All of the patients were diagnosed histologically according to World Health Organization classification criteria by expert hematopathologists, and had to have 1 or more of ^18^F-FDG avid measurable lesions in pretreatment PET/CT scan. ^18^F-FDG avid lesion was defined as having higher ^18^F-FDG activity than the surrounding tissue on visual analysis. Patients were staged according to the Ann Arbor Staging System and classified based on International Prognostic Index (IPI) and MALT-IPI (age, stage, lactate dehydrogenase)^22^. Patients were treated with 6 cycles of immunochemotherapy consisting of R-CVP (rituximab, cyclophosphamide, vincristine, prednisolone), R-CHOP (rituximab, cyclophosphamide, doxorubicin, vincristine, prednisolone), R-B (rituximab, bendamustine; usually every 4 weeks) in standard doses every 3 weeks. The interim response assessment was conducted after 2 or 3 cycles of immunochemotherapy, while the final response was assessed 1 month after completion of the first-line treatment. Follow-up restaging was done every 3–6 months during the first year and every 6–12 months thereafter. All patients were eligible for inclusion after the protocol was approved by the Institutional Review Board of Chonnam National University Hwasun Hospital and the Institutional Review Board of each participating institution in accordance with the Declaration of Helsinki.

### Procedures and assessment of PET/CT

PET/CT was performed at each hospital using a dedicated combined PET/CT scanner that considered the technical characteristics of each center. The patients fasted for 6–8 h prior to the intravenous administration of ^18^F-FDG (4.1–7.4 MBq per body weight) to ensure a serum glucose level below 180 mg/dL. At 60 ± 10 min after the intravenous ^18^F-FDG administration, a single-spiral CT scan [120–140 kV, automated from 10 to 160 mA, a 1–5 mm slice thickness, and a rotation time of 0.7–0.8 s] and emission scan extending from the base of the skull to the proximal thighs with a 15.0 cm–20.0 cm axial field of view acquired in 2.5–4.0 min per bed position were consecutively performed. The images were reconstructed using conventional reconstruction (OSEM) or combination of OSEM and the following algorithms such as time-of-flight (TOF) and point-spread-function (PSF). The details of the PET/CT scanners used at each hospital are listed in Supplementary material Appendix 1. The acquisition and reconstruction parameters of PET/CT scan were different between participating institutions. However, PET/CT was performed with one of dedicated PET/CT scanner in each hospital and follow-up scan was done with the same camera and same reconstruction algorithm as used for the initial scan. The initial and interim staging CT and PET/CT scans were assessed according to Lugano classification^2^. The PET/CT scans were read independently by each participating center’s nuclear medicine physicians. The interim PET/CT scans were compared with the baseline PET/CT scans according to both visual assessment using Deauville criteria^23^ and quantitative assessment of FDG uptake using the percentage of maximal standardized uptake value (SUV) reduction (ΔSUVmax) between the initial and interim PET/CT scans. Among the patients who achieved a score 3 or 4 Deauville response on the interim PET/CT, the visual assessment was rechecked by another independent nuclear medicine physician. On axial, coronal, or sagittal co-registered PET/CT slices, simple circular regions of interest were corrected for body weight according to the following standard formula: mean region of interest activity (MBq/mL)/[injected dose (MBq)/body weight (kg)]^24^. The ΔSUVmax was calculated as follows: ΔSUVmax (%) = 100 × [SUVmax (initial) – SUVmax (interim)]/SUVmax (initial). For each PET/CT scan, SUVmax was defined as the highest SUV among all hypermetabolic tumor lesions. Regarding the extranodal involvement assessment, especially in cases of gastrointestinal involvement, PET/CT was considered positive when if the focal uptake of FDG was more intense than that of the surrounding tissue. An endoscopic examination was performed if the distinction between the lymphoma lesion and normal tissue was unclear. As for bone marrow (BM) involvement, focal areas of increased FDG uptake in anywhere in the BM were considered positive. However, PET/CT is known to be less sensitive to detect BM involvement in indolent lymphoma, all of the patients were taken BM biopsy.

### Statistical analysis

PFS was a primary endpoint in the evaluation of the prognostic significance of interim PET/CT. PFS was defined as the time from treatment to disease progression or death of any cause, while overall survival (OS) was defined as the time from treatment to death from any cause. The Kaplan–Meier method was used to estimate the PFS and OS, and the survival curves were compared using a log-rank test. To evaluate the optimal cutoff value of SUVmax for predicting PFS, receiver-operating characteristic curve (ROC) analysis was performed. The estimate of the relative risk of event and its 95% confidence interval (CI) for PFS and OS were assessed by univariate and multivariate analyses using a Cox proportional hazard model. All of the statistical computations were performed using SPSS software (ver. 21; SPSS Inc., Chicago, IL, USA). The *P* values < 0.05 were considered significant in all of the analyses.

### Ethical approval

All procedures performed in studies involving human participants were in accordance with the ethical standards of the institutional and/or national research committee and with the 1964 Helsinki declaration and its later amendments or comparable ethical standards.

### Informed consent

Informed consent was obtained from all individual participants or next of kin of dead patients included in the study.

## Results

### Patient characteristics

The clinical characteristics of the 146 enrolled patients are summarized in Table 1. One hundred and twenty-five patients (85.6%) were diagnosed with extranodal MZL, 18 patients (12.3%) with nodal MZL, and 3 patients (2.1%) with splenic MZL. One hundred and twenty-one patients (82.9%) presented with advanced stage disease (III–IV), while 25 patients (17.1%) with stage II disease with bulky mass or rapid disease progression. Patients were classified according to IPI and MALT-IPI risk stratification^22^. OS of the enrolled patients was well stratified according to IPI and MALT-IPI risk groups, although the median OS was not reached (IPI, *p* = 0.043; MALT-IPI, *p* = 0.001). The most commonly involved extranodal site was the gastrointestinal tract (26.0%), followed by the lung (20.5%) and ocular area (17.8%). The majority of the patients were administered R-CVP (90.4%) as the first-line therapy.

**Table 1:** Patient’s clinical characteristics (N = 146).

Variables	No. of patients	%
Median age, years (range)	59.5 (28.0–84.0)	
Sex		
Male	85	58.2
Female	61	41.8
Histologic subtypes		
Nodal MZL	18	12.3
MALT lymphoma	125	85.6
Splenic MZL	3	2.1
ECOG PS		
0–1	131	89.7
≥ 2	15	10.3
Ann Arbor stage		
II	25	17.1
III–IV	121	82.9
Increased LDH	34	23.3
Bone marrow involvement	51	34.9
Extranodal site involvement	128	87.7
Extranodal site > 1	51	34.9
Extranodal site		
Ocular	26	17.8
Lung	30	20.5
GI tract	38	26.0
Spleen	17	11.6
Bone	3	2.1
IPI risk group		
Low (0–1)	43	29.5
Low-intermediate (2)	51	34.9
High-intermediate (3)	36	24.7
High (4–5)	16	11.0
MALT-IPI		
Low (0)	21	14.4
Intermediate (1)	85	58.2
High (2–3)	39	26.7
First-line treatment		
R-CVP	132	90.4
R-CHOP	9	6.2
R-B	5	3.4

*No.* number, *ECOG PS* Eastern Cooperative Oncology Group Performance Status, *LDH* lactate dehydrogenase, *GI* gastrointestinal, *IPI* International Prognostic Index, *MALT* mucosa-associated lymphoid tissue.

### Response evaluation of interim PET/CT and survival outcomes

At the time of the analysis, 45 patients (30.8%) had relapsed or progressed and 12 patients (8.2%) died. With a median follow-up of 42.0 months, the probability of 4-year PFS was 62.6% (95% CI 52.6–72.6), and the probability of 4-year OS was 91.0% (95% CI 85.5–96.5). According to DS of the interim PET/CT, 39 patients (26.7%) had a score of 1, 38 patients (26.0%) had a score of 2, 27 (18.5%) patients had a score of 3, 35 patients (24.0%) had a score of 4, and 7 patients (4.8%) had a score of 5. Treatment was not changed according to the interim PET/CT unless definite disease progression was found in interim PET/CT. One patient with DS 5 who had disease progression at the time of interim PET/CT assessment received palliative radiation therapy. The median SUVmax of the lesion was 4.9 (range, 0.8–23.5) on the initial PET/CT, while the median reduction rate of the SUVmax (ΔSUVmax) was 47.1% (range, -77.3 to 95.3) from the diagnosis to the interim PET/CT. The optimal cutoff value of the ΔSUVmax for disease progression was 59.8% according to the ROC analysis (sensitivity, 48.7%; specificity, 64.7%; area under the curve, 0.532; 95% CI 0.444–0.618). Using the cutoff value of ΔSUVmax, 52 patients (35.6%) were classified as good responders (ΔSUVmax ≥ 59.8), while 81 patients (55.5%) were classified as poor responders (ΔSUVmax < 59.8).

**Figure 1:**
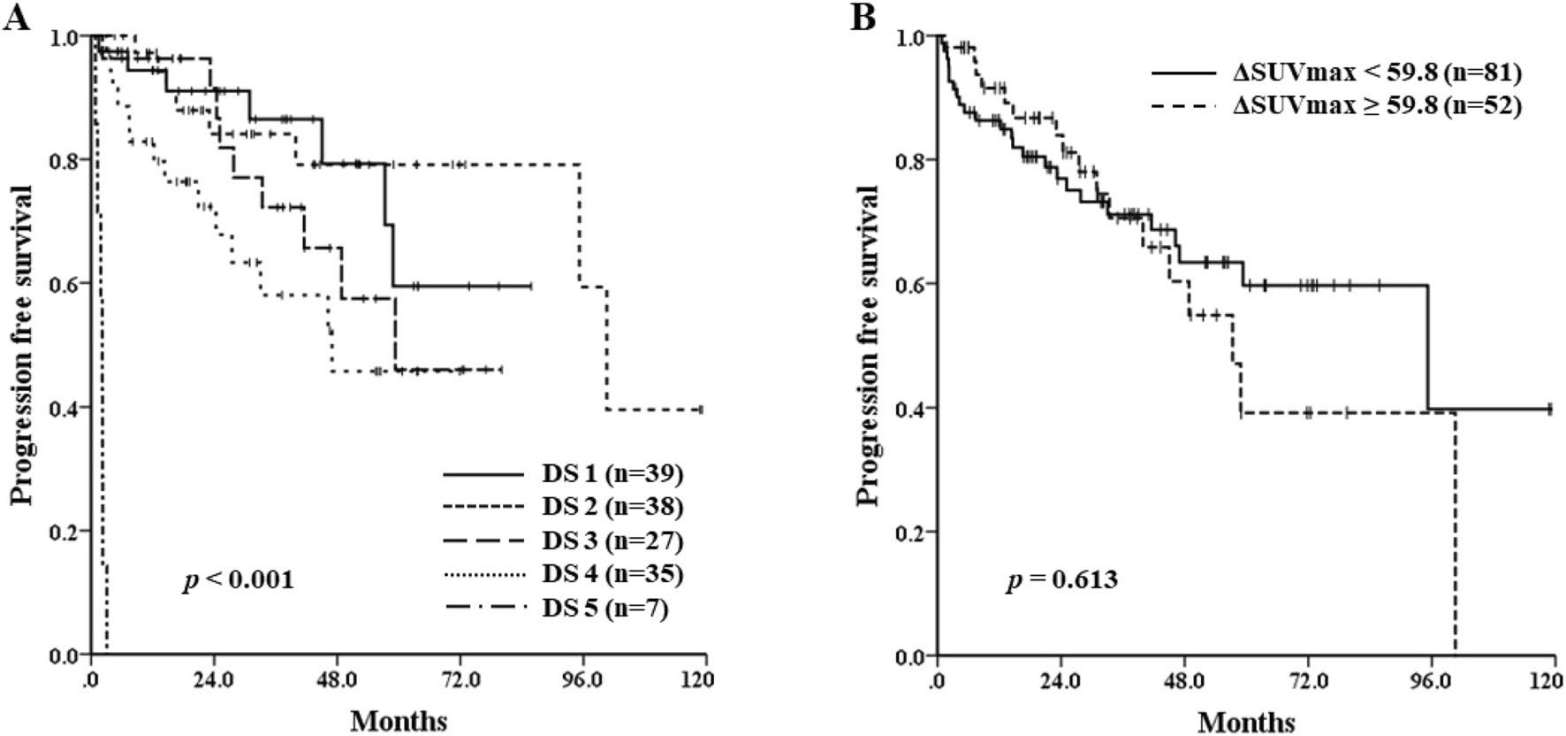
Progression-free survival of the patients according to the visual assessment of interim ^18^F-FDG PET/CT response using the Deauville 5-point scale after 2–3 cycles of immunotherapy (**A**) and quantitative assessment using ΔSUVmax (**B**).

**Figure 2:**
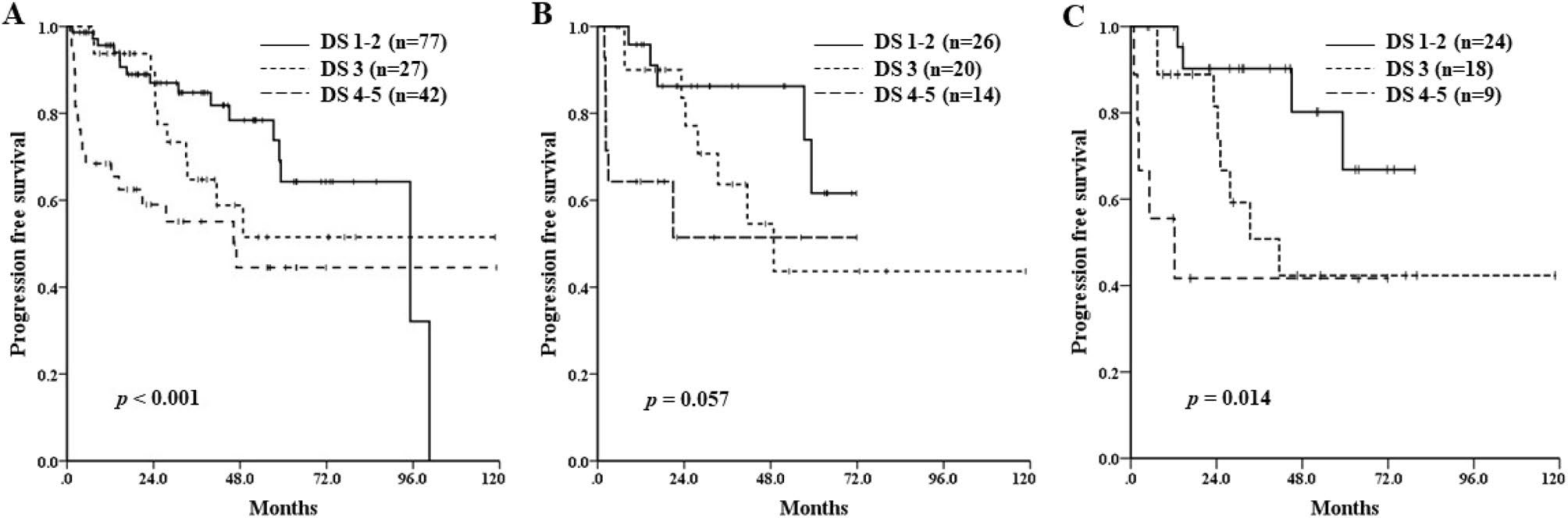
Progression-free survival of all patients (**A**), patients with gastrointestinal tract or lung involvement (**B**), and patients with the involvement of more than 2 extranodal sites (**C**) based on the visual assessment of interim ^18^F-FDG PET/CT response using the Deauville 5-point scale after 2–3 cycles of immunotherapy.

PFS differed significantly by DS (median PFS of each grade: not reached vs. 100.6 months vs. 59.3 months vs. 47.0 months vs. 2.1 months, respectively; *p* < 0.001) (Fig. 1A). However, regarding the SUV-based assessment, ΔSUVmax could not predict the survival outcomes. Median PFS was 95.2 months for poor responders (ΔSUVmax < 59.8) and 57.3 months for good responders (ΔSUVmax ≥ 59.8) (*p* = 0.613) (Fig. 1B). In a subgroup analysis of 24 patients with an initial PET/CT SUVmax ≥ 10, median PFS was better in good responders, although not statistically significant (48.8 months vs. 7.4 months; *p* = 0.06). The majority of the patients (87.7%) had the involvement of 1 or more extranodal sites at diagnosis, while 51 patients (34.9%) had the involvement of more than 2 extranodal sites. The difference of PFS between the patients with interim DS 1–2 and DS 3–5 was significant, and patients with interim DS of 3 had relatively worse PFS than those with DS 1 or 2 on the interim PET/CT scan, especially in cases with gastrointestinal tract or lung involvement or with the involvement of more than 2 extranodal sites (Figs. 2 and Supplementary Appendix 2). At the end of treatment, using the PET/CT response assessment, 106 patients (72.6%) achieved a complete response (CR), 28 (19.2%) achieved a partial response (PR), 6 (4.1%) achieved stable disease (SD), and 6 (4.1%) achieved progressive disease (PD). All patients with PD except one who refused further chemotherapy received salvage chemotherapy and 1 patient with SD on end-of-treatment PET/CT scan underwent palliative radiotherapy. Patients who had achieved objective responses did not receive any other treatment and were treated again when they obviously relapsed or progressed. There was no significant difference in OS according to the DS on interim PET/CT (DS 1–2 vs. DS 3 vs. DS 4–5, *p* = 0.101).

The univariate and multivariate analyses of PFS are summarized in Table 2. A poor PS (PS ≥ 2), increased lactate dehydrogenase level, high-intermediate or high IPI risk, no achievement of DS 1–2 on the interim PET/CT were poor prognostic factors on the univariate analysis of PFS. In the multivariate analysis, no achievement of DS 1–2 on the interim PET/CT was significantly associated with an inferior PFS (hazard ratio, 2.154; 95% confidence interval, 1.071–4.332; *p* = 0.031). However, interim PET/CT response was not a significant prognostic factor and, MALT-IPI was the only strong prognostic factor of OS on the multivariate analysis.

**Table 2:** Factors affecting progression free survival in all patients.

Variables	Univariate			Multivariate		
	HR	95% CI	*p *value	HR	95% CI	*p *value
Age > 70	1.084	0.454–2.589	0.856			
Gender (female)	0.713	0.388–1.308	0.274			
ECOG PS ≥ 2	2.809	1.295–6.093	0.009	0.963	0.316–2.931	0.947
Increased LDH	2.280	1.209–4.298	0.011	1.497	0.543–4.128	0.435
EN involvement > 1	1.293	0.508–3.290	0.590			
BM involvement	1.032	0.557–1.911	0.920			
IPI high-intermediate and high	1.730	0.958–3.124	0.069			
MALT-IPI 3	3.534	0.854–14.626	0.082			
ΔSUVmax < 59.8	1.173	0.631–2.181	0.613			
No achievement of DS 1–2 in interim PET/CT	2.103	1.140–3.877	0.017	2.154	1.071–4.332	0.031

*HR* hazard ratio, *CI* confidence interval, *ECOG PS* Eastern Cooperative Oncology Group Performance Status, *LDH* lactate dehydrogenase, *EN* extranodal, *BM* bone marrow, *IPI* International Prognostic Index, *MALT* mucosa-associate lymphoid tissue, *SUV* standardized uptake value, *DS* Deauville score.

## Discussion

There have been controversies about the clinical usefulness of response assessment using PET/CT for MZL. This study aimed to evaluate the prognostic relevance of the interim PET/CT scan for MZL by current standard response criteria, and the results suggest that the interim PET/CT scan can predict the clinical outcomes of advanced MZL patients who were treated with immunochemotherapy. Achievement of DS 1–2 in the interim PET/CT was significantly associated with prolonged PFS. In addition, Patients who achieved DS 1–2 showed longer PFS than those who achieved DS 3. It can be probably explained because of extranodal involvement sites was overestimated due to the physiologic uptake of the surrounding tissue. However, response assessment using ΔSUVmax could not predict the survival outcome since the initial SUVmax of tumor lesion was low.

To strengthen the uniformity of the cohort, we excluded patients with early stage disease who did not undergo any treatment or received only involved-field radiation therapy or who received cytotoxic chemotherapy without rituximab. Regarding the PET/CT analysis, a visual assessment using DS has been the preferred measurement since the First International Workshop on Interim PET Scan in Lymphoma in 2009 in Deauville, France^23^. The visual assessment of the interim PET/CT response had a prognostic impact (Fig. 1A). A DS grade of 1–3 generally represents a complete metabolic response, but there have been some arguments about the clinical meaning of a grade 3 response^25–27^. In this study, the median PFS of the patients with an interim PET/CT DS 1–2 was longer than that of the patients with interim PET/CT DS 3 (Fig. 2A). The cause of such a difference in survival results could be explained by the extranodal involvement of the MZL. As shown in Fig. 2, the subgroup of patients with the involvement of more than 2 extranodal sites and those with gastrointestinal tract or lung involvement showed a lower median PFS in the interim PET/CT DS 3 group than in the DS 1–2 group. These results suggest that the achievement of a DS 1–2 could predict a better prognosis than a DS 3.

However, the semi-quantitative method using SUVmax failed to predict the survival outcome in this study. This might be caused by the low baseline SUVmax (median 4.9). Similar results were seen in previous studies of diffuse large B-cell lymphoma. When baseline SUVmax was low, a target ΔSUVmax can be lower than the cutoff value^28,29^. Such false-positive results occurred when the baseline SUVmax was less than 10 in the LNH 2007-3B trial^30^. The subgroup analysis of our study patients who had a baseline SUVmax greater than 10, which showed prominent PFS prolongation in good responders (ΔSUVmax ≥ 59.8), supports that explanation although the PFS benefit was not statistically significant. Further research with a larger number of patients is needed to confirm the usefulness of SUV based response assessment in MZL.

This retrospective study has several limitations. First, it was a multicenter study and PET/CT scans were obtained in 13 different institutions, so the PET/CT instrument and imaging protocol such as image acquisition and reconstruction was unstandardized and this might has influenced the results. DS and SUVmax were not centrally determined, although the PET/CT image was reviewed by experienced physicians at each institution. The SUV reliability can be influenced by many factors such as blood glucose level, technological characteristics, partial volume effect, injected dose and decay of radiotracer^31^. However, recent studies suggest that PET/CT response assessments using DS have small interobserver differences^32,33^. Moreover, an absolute SUVmax can vary among institutions, but the reduction rate in SUVmax showed high interobserver reproducibility in several studies^34,35^. Second, nodal involvement remains unclear. Nodal MZL is known to have much higher ^18^F-FDG avidity than primary MALT lymphoma (100% vs. 54%), which might affect the PET/CT response assessment results^36^. Besides, only 3 splenic MZL patients were included in this study, so it is difficult to clarify the role of the interim PET/CT in this subgroup. Further prospective studies with larger numbers of patients are warranted to define the clinical impact of interim PET/CT in each MZL histological subgroup. In addition, this study included patients with advanced stage disease or stage II with aggressive characteristics, so the results cannot represent all patients with MZL. However, this study is the first to demonstrate the prognostic impact of interim ^18^F-FDG PET/CT response assessment using DS in a relatively large cohort.

In conclusion, DS–based interim PET/CT response assessment is useful for predicting the survival outcomes of patients with MZL treated with rituximab, whereas the semi-quantitative assessment based on ΔSUVmax had no prognostic impact.

## Supplementary information

Supplementary information

## Data Availability

The datasets generated during and/or analyzed during the current study are not publicly available.
