# Supplementary material for: Prognostic significance of interim PET/CT response for the treatment of advanced-stage marginal zone lymphoma in the post-rituximab era
Source: Sci Rep. 2020 Jul 15;10:11649. doi: 10.1038/s41598-020-68310-w (PMC7363857; doi:10.1038/s41598-020-68310-w)

**Supplement information**

**Prognostic significance of interim PET/CT response for the treatment of advanced-stage marginal zone lymphoma in the post-rituximab era**

Ga-Young Song^1^, Sang Eun Yoon^4^, Seok Jin Kim^4^, Jin Seok Kim^5^, [Youngil Koh](https://www.ncbi.nlm.nih.gov/pubmed/?term=Koh%20Y%5BAuthor%5D&cauthor=true&cauthor_uid=28675026)^6^, Joon-Ho Moon^7^, [Sung Yong Oh](https://onlinelibrary.wiley.com/action/doSearch?ContribAuthorStored=Oh%2C+Sung+Yong)^8^, Ho Sup Lee^9^, Ho-Jin Shin^10^, Young Rok Do^11^, Won Sik Lee^12^, Dae sik Kim^13^, Yong Park^14^, Ho-Young Yhim^2,3*^, and Deok-Hwan Yang^1*^

^1^Chonnam National University Hwasun Hospital, Hwasun, Jeollanam-do; ^2^Jeonbuk National University Medical School, Jeonju, Jeollabukdo; ^3^Research Institute of Clinical Medicine of Jeonbuk National University-Biomedical Research Institute of Jeonbuk National University Hospital, Jeonju; ^4^Sungkyunkwan University School of Me dicine, Seoul; ^5^Yonsei University College of Medicine, Seoul; ^6^Seoul National University Hospital, Seoul, Korea; ^7^Department of Hematology/Oncology, Kyungpook National University Hospital, Kyungpook National University School of Medicine, Daegu; ^8^Dong-A Medical Center, Busan; ^9^Kosin University Gospel Hospital, Busan; ^10^Division of Hematology-Oncology, Department of Internal Medicine, Medical Research Institute, Pusan National University Hospital, Pusan National University School of Medicine; ^11^Keimyung University Dongsan Medical Center, Daegu; ^12^Busan Paik Hospital, Inje University, Busan; ^13^Korea University Guro Hospital, Seoul; ^14^Division of Hematology/Oncology, Department of Internal Medicine, Korea University School of Medicine Anam Hospital, Seoul, Republic of Kore

**Running Title:** Response assessment of interim PET/CT in marginal zone lymphoma

*These authors contributed equally to this study.

**Co-corresponding authors:**

**Deok-Hwan Yang*, M.D., Ph.D.**

Department of Hematology/Oncology

Chonnam National University Hwasun Hospital, School of Medicine, Chonnam National University

322 Seoyang-ro, Hwasun-eup, Hwasun-gun, Jeollanam-do, 58128, Republic of Korea

[Tel: +82-61-379-7636](Tel:+82-61-379-7636)

Fax: +82-61-7628

E-mail: [drydh1685@hotmail.com](mailto:drydh1685@hotmail.com)

**Ho-Young Yhim*, M.D., Ph.D.**

Department of Internal Medicine
Jeonbuk National University Medical School

Research Institute of Clinical Medicine of Jeonbuk National University-Biomedical Research Institute of Jeonbuk National University Hospital

20 Geonji-ro, Deokjin-gu, Jeonju, 54907, Republic of Korea
Phone: +82-63-250-2523
Fax: +82-63-255-1609
E-mail: yhimhy@jbnu.ac.kr

**Appendix 1: List of dedicated PET/CT scanners, and participating centers**

1) Discovery ST PET-CT scanner (GE Healthcare, Milwaukee, WI, USA), Discovery 600 PET-CT scanner (GE Healthcare). Chonnam National University Hwasun Hospital, Jeollanam-do, South Korea **(22 patients)**.

2) Biograph 16 PET-CT scanner (Siemens Medical Solutions, Knoxville, TN, USA), Biograph TruePoint 40 PET-CT scanner (Siemens Medical Solutions). Chonbuk National University Hospital, Jeonju, South Korea **(16 patients)**.

3) Discovery LS PET-CT scanner (GE Healthcare), Discovery STE PET-CT scanner (GE Healthcare). Samsung Medical Center, Seoul, South Korea **(18 patients)**.

4) GE advance PET scanner (GE, Milwaukee, WI, USA), Philips Allegro PET system (Allegro, Philips-ADAC medical systems, Cleveland, OH). Yonsei University College of Medicine, Seoul, South Korea **(5 patients)**

5) GE advance PET scanner (GE, Milwaukee, WI, USA), Gemini TF scanner (Philips Healthcare, Cleveland, OH, USA). Seoul National University College of Medicine, Seoul, South Korea **(20 patients)**

6) Discovery ST PET-CT scanner (GE Healthcare), Biograph 6 PET-CT scanner (Siemens Medical Solutions). Kyungpook National University Hospital, Daegu, South Korea **(4 patients)**.

7) Gemini TF 16 (Philips Medical Systems), Discovery 710 (GE Healthcare). Dong-A University Hospital, Busan, South Korea **(10 patients)**.

8) Biograph Duo PET/CT scanner (Siemens Healthcare, Erlangen, Germany), Biograph 16 PET/CT scanner (Siemens Healthcare, Erlangen, Germany). Kosin University Gospel Hospital, Busan, South Korea **(11 patients)**

9) Biograph 40 PET-CT scanner (Siemens Medical Solutions), Gemini PET-CT scanner (Philips Medical System). Pusan National University Hospital, Busan, South Korea **(6 patients)**.

10) Discovery STE PET-CT scanner (GE Healthcare). Keimyung University Dongsan medical center, Daegu, South Korea **(1 patients)**

11) Discovery STE PET-CT scanner (GE Healthcare). Inje University Busan Paik Hospital, Busan, South Korea **(7 patients)**.

12) Gemini TF 16 (Philips Medical Systems). Korea University Guro Hospital, Seoul, South Korea **(5 patients)**.

13) Gemini TF (Philips Medical Systems, Cleveland, OH, USA), Gemini PET-CT scanner (Philips Medical System). Korea University Anam Hospital, Seoul, South Korea **(21 patients)**.

**Appendix 2: Progression-free survival of Deauville score 1-2 versus 3-5 in all patients (A), patients with gastrointestinal tract or lung involvement (B), and patients with the involvement of more than 2 extranodal sites (C) based on the visual assessment of interim ^18^F-FDG PET/CT response after 2–3 cycles of immunotherapy.**


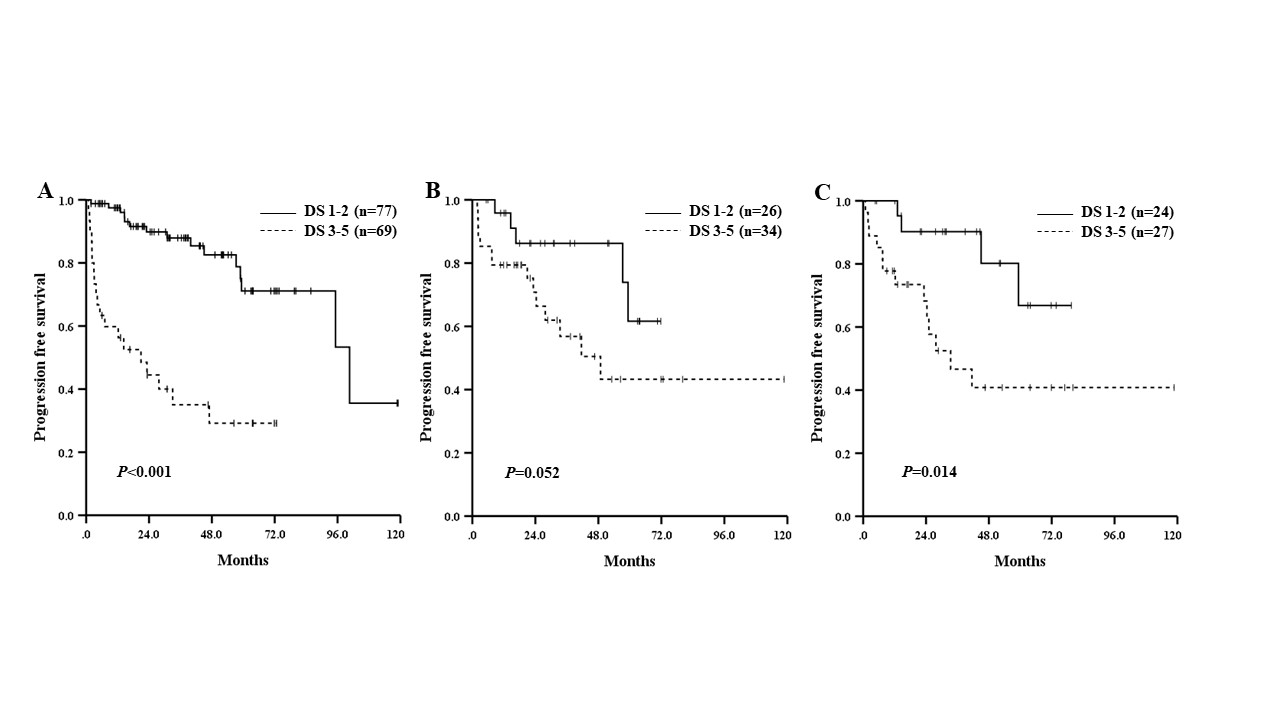

Supplement: Supplementary file 1 — Supplementary information [file 41598_2020_68310_MOESM1_ESM.docx]
